# Water Exchange from the Buried Binding Sites of Cytochrome P450 Enzymes 1A2, 2D6, and 3A4 Correlates with Conformational Fluctuations

**DOI:** 10.3390/molecules29020494

**Published:** 2024-01-19

**Authors:** Olgun Guvench

**Affiliations:** Department of Pharmaceutical Sciences and Administration, School of Pharmacy, Westbrook College of Health Professions, University of New England, 716 Stevens Avenue, Portland, ME 04103, USA; oguvench@une.edu

**Keywords:** cytochrome P450, CYP, 1A2, 2D6, 3A4, binding site, conformation, flexibility, water, molecular dynamics

## Abstract

Human cytochrome P450 enzymes (CYPs) are critical for the metabolism of small-molecule pharmaceuticals (drugs). As such, the prediction of drug metabolism by and drug inhibition of CYP activity is an important component of the drug discovery and design process. Relative to the availability of a wide range of experimental atomic-resolution CYP structures, the development of structure-based CYP activity models has been limited. To better characterize the role of CYP conformational fluctuations in CYP activity, we perform multiple microsecond-scale all-atom explicit-solvent molecular dynamics (MD) simulations on three CYP isoforms, 1A2, 2D6, and 3A4, which together account for the majority of CYP-mediated drug metabolism. The MD simulations employ a variety of positional restraints, ranging from keeping all CYP atoms close to their experimentally determined coordinates to allowing full flexibility. We find that, with full flexibility, large fluctuations in the CYP binding sites correlate with efficient water exchange from these buried binding sites. This is especially true for 1A2, which, when restrained to its crystallographic conformation, is unable to exchange water between the binding site and bulk solvent. These findings imply that, in addition to crystal structures, a representative ensemble of conformational states ought to be included when developing structure-based CYP activity models.

## 1. Introduction

Human cytochrome P450 enzymes (CYPs) are a major contributor to the metabolism of small-molecule pharmaceuticals (drugs) and metabolize ~75% of FDA-approved drugs [1,2]. CYPs accomplish this action by catalyzing oxidation reactions that convert hydrophobic substrates to hydrophilic products [3]. This conversion alters the pharmaceutical activity, leading to products that can be less or more active than the substrates [4], and generally favors the elimination of compounds from the body owing to either the direct enhancement of water solubility or the facilitation of subsequent conjugation reactions that further enhance water solubility [5]. In addition to drug metabolism by CYPs, there also exist the possibilities of drugs acting to inhibit or to induce CYP activity [6].

Important opportunities for the application of computational approaches in drug discovery and design are the prediction of the drug-specific impacts of and impacts on CYP activity. Experimental approaches typically entail the extraction of CYP-rich microsomes from hepatic tissue for assaying candidate drug molecules [7,8]. By reducing the need for such experimental assays, predictive computational models can optimize the time and materials costs associated with determining CYP activity in relation to a drug or drug candidate.

Two major categories of computational modeling for CYP activity/inhibition are “ligand-based” and “structure-based” [9]. Ligand-based models are developed using known structures and activities of drugs, which serve as a training set without consideration of the structure of the CYP enzymes themselves. In contrast, structure-based methods directly use the three-dimensional structures of CYP enzymes. “Machine learning”， which has typically been used to support ligand-based modeling [9,10], has the capacity to simultaneously incorporate aspects associated with ligand-based and structure-based approaches within a single model [11], and therefore may be considered a third category. The success of ligand-based modeling is impressive, with the caveat that a limitation or bias is necessarily imposed by the training set. Such limitation/bias is relevant when attempting to evaluate compounds that do not have substantial chemical similarity to compounds included in the training, leading to missed predictions, or conversely, when the training set is very structurally diverse, leading to a general model that cannot be used to fine-tune structural changes during lead optimization [12]. Structure-based methods are conceptually attractive, as they do not suffer this training-set-associated limitation/bias and can also take into account drug chirality, unlike ligand-based models trained using 2D chemical structures [9]. However, the development of structure-based models has proven to be a challenge despite the availability of experimental atomic-resolution structures for CYPs [13]. Factors contributing to this challenge are the range of different human CYPs and their differing substrate binding sites; the buried nature of CYP binding sites; and the plasticity of the binding site of a given CYP, which can entail different binding site conformations that enable binding of structurally diverse substrates [14].

Toward addressing these three factors, we apply all-atom explicit-solvent molecular dynamics (MD) simulations to determine the binding site properties of three distinct CYPs, 1A2, 2D6, and 3A4, because these three CYP isoforms together metabolize ~50% of the CYP-metabolized drugs that are approved or in development [2]. Our analysis of the MD data focuses on the hydration properties of the CYP binding sites, with the aim of determining the extent of binding-site hydration in the absence of a ligand and the rate of exchange of water molecules between the binding site and bulk solvent, along with the dependence of these properties on the flexibility of the CYP protein. We find that restraining CYP atoms to their positions as solved using X-ray crystallography can slow down binding site water exchange with the bulk solvent, and very dramatically in the case of 1A2, whereas the full protein flexibility observed in non-restrained MD enables the efficient exchange of water molecules. Additionally, both the average volumes of the 1A2, 2D6, and 3A4 binding sites and the observed fluctuations in these volumes tend to be smaller in restrained simulations relative to non-restrained simulations, suggesting a link between enhanced structural fluctuations and water exchange. Our findings support the use of conformational ensembles determined using non-restrained MD simulations in developing structure-based models for predicting the binding of small molecules to CYPs.

## 2. Results and Discussion

### 2.1. CYP Binding Site Residues Are Flexible

**Figure 1:**
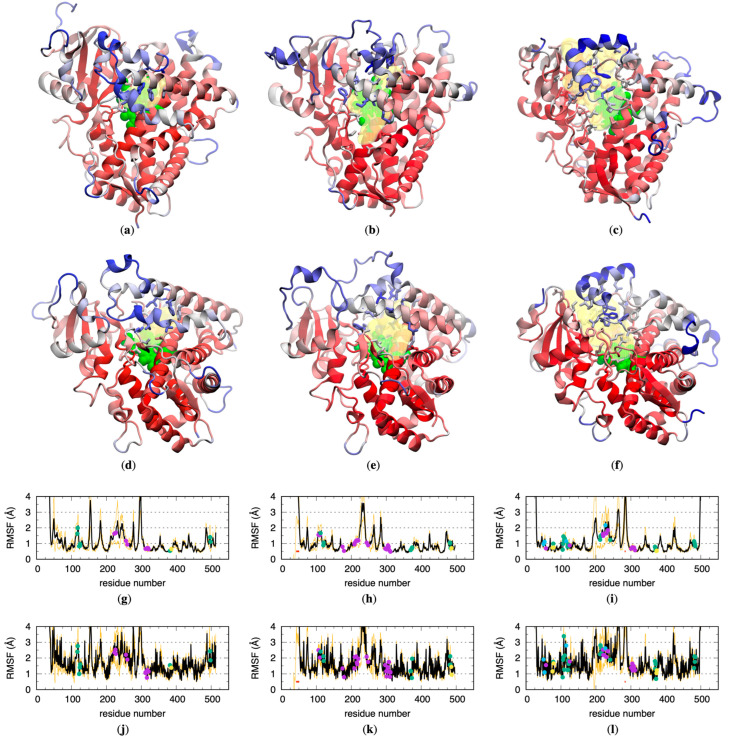
CYP crystallographic binding sites and their flexibility in non-restrained MD simulations. Molecular graphics of the 1A2, 2D6, and 3A4 crystal structures used as MD starting conformations are drawn from (**a**–**c**) the “distal face” and (**d**–**f**) the “side view” perspectives [16], with the binding site volume as determined using fpocket shown as a transparent yellow surface, the associated binding site residue (see Table 1) sidechains as sticks, and the heme group as green van der Waals spheres. The protein cartoon representation is colored according to data in the corresponding C*^α^* root-mean-squared fluctuation (RMSF) graph (**g**–**i**), with increasing values from red through white and on to blue. Binding site residue sidechains are likewise colored according to data in the corresponding sidechain RMSF graph (**j**–**l**). Binding site residues are noted as points in (**g**–**l**) and are colored according to the STRIDE [17] secondary structure computed from the corresponding crystal structure conformation (AlphaHelix = purple, 310Helix = blue, Strand = yellow, Coil/Turn/Bridge = green), and residues missing in the crystal structures are noted with a red bar at *y* = 0.5 Å. RMSFs are averaged across all non-restrained MD trajectories for a given CYP (see Section 4) and are drawn as black lines, with orange lines at +/− one standard deviation. For clarity, the range of color red through blue for the molecular graphics is determined using the minimum overall RMSF for the minimum value and the maximum binding site residue RMSF for the maximum value from the corresponding graph.

The CYP 1A2, 2D6, and 3A4 crystal structure conformations all have buried binding sites characterized by a large pocket similarly located within the interiors of the proteins and defined by the heme moiety on one side. Fpocket [15] analysis (see Section 4) of these crystal structures identifies many pockets for each structure, although, upon visual inspection, for each protein crystal structure there is one obvious pocket corresponding to the binding site (Figure 1a–f). Per fpocket, the respective volumes for the 1A2, 2D6, and 3A4 pockets are 512 Å^3^, 1019 Å^3^, and 2206 Å^3^, and these pockets are defined by the heme moiety plus 20, 34, or 46 amino acid residues (Table 1). In addition to the contrasting volumes and numbers of associated residues, the pockets for 2D6 and for 3A4 both contain portions that connect the binding sites to the exterior of the protein, whereas the pocket for 1A2 does not.

All-atom explicit-solvent MD simulations started from the crystallographic conformations showed that binding site residues include atoms with high flexibility. To minimize bias introduced by equilibration of the solvent causing perturbation of the crystallographic conformations, each MD simulation entailed five successive 250 ns stages that created a single, continuous 1250 ns trajectory: Stage 1 had strong positional restraints on all non-hydrogen protein atoms, Stage 2 had weak positional restraints on all non-hydrogen protein atoms, Stage 3 had weak positional restraints on C*^α^* protein atoms only, and Stage 4.1 and Stage 4.2 had no positional restraints on any individual atoms (see Section 4). As expected, high root-mean-squared fluctuation (RMSF) values of the protein backbone C*^α^* and sidechain atoms from the non-restrained (Stage 4.1 and 4.2) portions of these trajectories were associated with loop and N-terminal regions of these proteins due to their lack of *α*-helical or *β*-strand secondary structures (Figure 1a–f). Interestingly, high RMSF values were also associated with those atoms in *α*-helical portions of the proteins that contained binding site residues (Figure 1a–l), in contrast with other *α*-helical portions of the proteins, which tended to have the lowest RMSF values. This high degree of flexibility in binding site residues with crystallographic secondary structures is presumably an enabling factor in allowing either substrate access to or product release from the 1A2, 2D6, and 3A4 binding sites, or in allowing the binding site for a given CYP to bind a diverse array of substrates.

### 2.2. Conformational Flexibility Is Required for Binding Site Water Access in CYP 1A2 but Not in CYP 2D6 or CYP 3A4

The restrained stages of the MD trajectories highlight the variable importance of protein flexibility in enabling access to the buried binding sites of CYP 1A2, 2D6, and 3A4. Because the current MD data are for systems without small molecule substrates, surveying binding site water molecule hydrogen-bond networks can provide a proxy for binding site accessibility from the bulk solvent, with the caveat that water molecules and drug molecules may use different routes to transit between the bulk solvent and a binding site [18,19,20]. 

**Figure 2:**
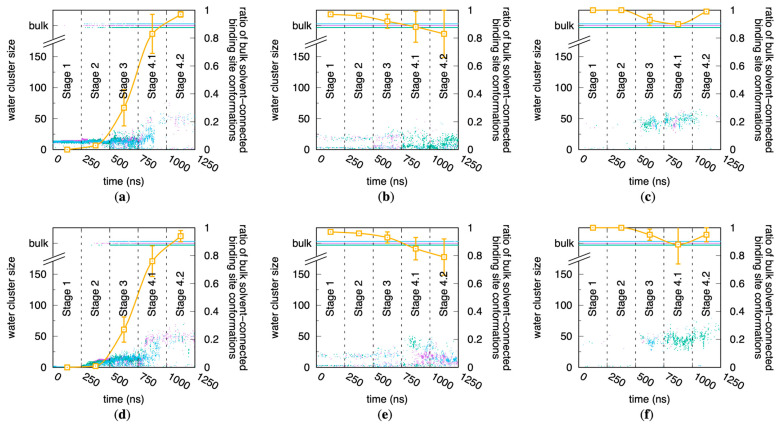
Connection of CYP binding sites to the bulk solvent as a function of increasing protein flexibility. Data are for 1A2, 2D6, and 3A4 using (**a**–**c**) the “2 Å” and (**d**–**f**) the “3 Å” solvation protocols (see Section 4). Purple/green/blue dots are the water cluster size time series data from triplicate MD simulations for each CYP/protocol combination, with water cluster size computed as described in Section 4; data for clusters connected to the bulk solvent are plotted at values of “bulk”, “bulk−”, and “bulk+” for clarity. The per-stage ratios of bulk solvent-connected binding site conformations, plotted in orange, are derived from these data, with ratios computed for each stage of each run and then averaged across the three runs; error bars are the standard deviation in the three-run average ratio for each stage. In a time series, a binding site conformation is considered to be bulk solvent-connected if its corresponding water cluster size = “bulk” (see Section 4).

In the case of CYP 1A2, the creation of a bulk solvent-connected hydrogen-bond network of water molecules is not possible when the protein is restrained to its crystallographic conformation during the Stage 1 MD. In Stage 1, an isolated cluster of water molecules exists adjacent to the heme iron. It is only with the introduction of full sidechain flexibility in Stage 3 of the CYP 1A2 simulations that a substantial ratio, ~30% (Figure 2a,d), of the MD snapshots evince a connected network composed of contiguous hydrogen-bonded water molecules spanning the binding site to the bulk solvent. Furthermore, it is only when CYP 1A2 is non-restrained in Stage 4.1 and Stage 4.2, and therefore allowed to sample its natural ensemble of conformational states, that the large majority of individual conformations (MD snapshots) contain this connected network (Figure 2a,d).

This same analysis for CYP 2D6 and CYP 3A4 reveals that, despite being homologs of 1A2, these two proteins in their crystal structure conformations can accommodate a connected network of water molecules between the binding site and bulk solvent (Figure 2b,e and c,f). The large majority of individual conformations across all of the MD stages for 2D6 and 3A4 have such a hydrogen-bonded water network, including Stage 1, in which CYP atom coordinates are restrained to their crystallographic values. In fact, this is the case for nearly all sampled conformations in Stage 1 and Stage 2 of the CYP 2D6 and 3A4 simulations, which is opposite the behavior of CYP 1A2. Interestingly, with the introduction of full protein flexibility in Stage 4.1, these two proteins sample conformations with binding sites that are cut off from the bulk solvent. It is tempting to speculate that these conformational fluctuations occurring in non-restrained MD may enhance catalysis by enabling full isolation of the binding site from the bulk solvent and stronger binding of substrates in poses with maximized protein–ligand interactions.

### 2.3. Binding Site Volumes Increase with Increased Protein Flexibility and Encompass Increasing Numbers of Water Molecules

**Figure 3:**
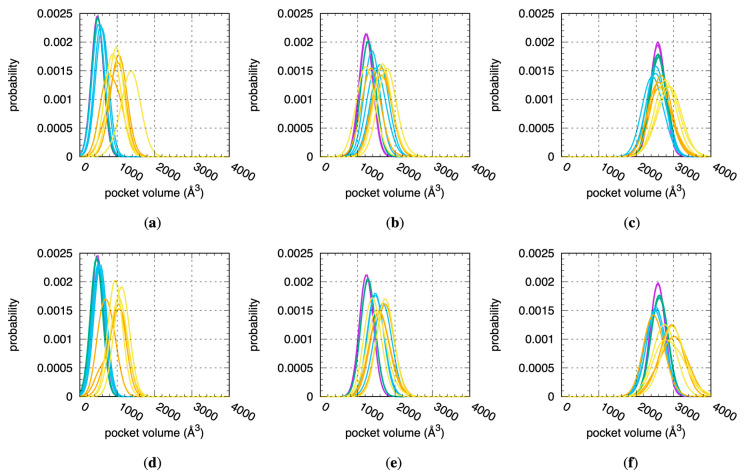
Distributions of fpocket-computed binding site volumes across all MD simulations. Data are for 1A2, 2D6, and 3A4 using (**a**–**c**) the “2 Å” and (**d**–**f**) the “3 Å” solvation protocols (see Section 4). Data for each distribution are from a single replicate for a single stage. Purple = Stage 1, green = Stage 2, blue = Stage 3, orange = Stage 4.1, yellow = Stage 4.2.

Increased CYP flexibility enables increased binding site volumes per fpocket analysis of the individual snapshots across all MD stages. To perform the analysis, the residues in Table 1 were used as the pocket definition input for fpocket. Distributions of the fpocket-computed volumes based on these pocket definitions and aggregated on a per-replicate and per-stage basis generally show a shift in distribution peaks to larger values in the progression from Stage 1 to Stage 4.x (Figure 3). The most dramatic change occurs in the 1A2 simulations, wherein the shift between the most probable volume sampled when the protein is restrained to its crystallographic conformation in Stage 1 to that sampled during the last, non-restrained stage of MD in Stage 4.2 is the difference between 500 Å^3^ and 1400 Å^3^—a 900 Å^3^, or 180%, increase (Figure 3a). The largest such increases for 2D6 and for 3A4 are 50% (1200 Å^3^ to 1800 Å^3^, Figure 3b) and 15% (2600 Å^3^ to 3000 Å^3^, Figure 3f), respectively. In addition to the increase in binding site pocket volumes, the non-restrained simulations exhibit increased fluctuations in binding site pocket volumes relative to the restrained simulations. This is most apparent when looking at the peak heights of the normalized probability distributions for these volumes: distributions from the Stage 4.x MD have significantly lower peaks and wider bases than those from the Stage 1 and 2 MDs for all three CYPs (Figure 3). Prior MD simulation of CYP 2D6 noted that the volume of its active site can fluctuate more than 50%, and this is true of the data from the present MD (Figure 3b,e) [21]. 

**Figure 4:**
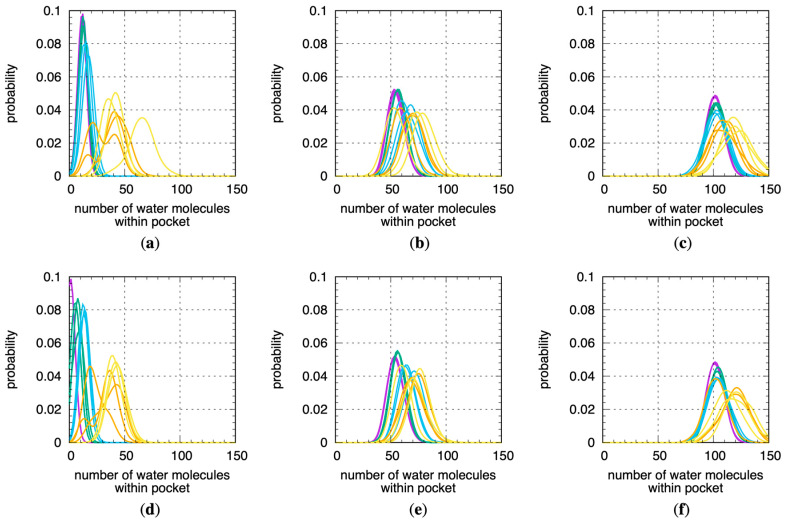
Distributions of the number of water molecules within fpocket-computed binding site volumes across all MD simulations. Data are for 1A2, 2D6, and 3A4 using (**a**–**c**) the “2 Å” and (**d**–**f**) the “3 Å” solvation protocols (see Section 4). Data for each distribution are from a single replicate for a single stage. Purple = Stage 1, green = Stage 2, blue = Stage 3, orange = Stage 4.1, yellow = Stage 4.2.

Not surprisingly, similar trends exist for the number of water molecules within the binding site pockets (Figure 4). For this analysis, for a given MD snapshot, a water molecule was taken to be within the binding site pocket volume if the oxygen atom of that water molecule was located within the sphere radius of any of the pocket alpha spheres computed using fpocket for that snapshot. The Stage 4.x MD data therefore imply that, in solution at physiological temperature, CYP 1A2, 2D6, and 3A4 in their substrate- or inhibitor-free forms sample well-hydrated binding site conformations that are enlarged relative to the crystallographic conformations. We note that a previous MD study found the 3A4 binding site to be occupied by 54–58 water molecules, whereas the present work puts this number at 110–120 (Figure 4c,f), and this may be explained by the 4 ns timescale of the production MD in that work versus the microsecond timescale MD here as well as by differences in how the binding site pocket was defined [22]. 

### 2.4. Protein Flexibility Is Especially Important for Binding Site Water Exchange in CYP 1A2

The equilibrium data from the MD simulations show that for CYP 1A2, protein flexibility is required to connect water molecules in the binding site with those in the bulk solvent via contiguous hydrogen-bond networks, but it is not required for CYP 2D6 and 3A4 (Figure 2). However, these data do not speak to the ability of water molecules within CYP binding sites to exchange with the bulk solvent external to the protein. That is to say, a particular water molecule within the binding site may participate in a hydrogen-bond network of water molecules connecting with the bulk solvent, yet this water molecule may still be effectively “stuck” within the binding site in terms of the timescales required for it to diffuse out into the bulk solvent. Correlation analysis of the residence of CYP binding site water molecules provides a kinetic perspective and clearly demonstrates that CYP flexibility correlates with the ability of water molecules in the binding site to move out into the bulk solvent and vice versa.

Correlation analysis was performed on a per-replicate, per-stage basis by comparing the identities of the water molecules within the binding site at time *t* and time *t* + Δ*t*. Each water molecule in the system was assigned a unique identifier, which it retained for the duration of the MD simulation. The correlation *C*(Δ*t*) is defined as:

$$C\left(\Delta t\right)≔{\langle \frac{n_{persistent}\left(t,t+\Delta t\right)}{n_{persistent}\left(t,t+\Delta t\right)+n_{transient}\left(t,t+\Delta t\right)}\rangle }_{t=0\;ns\ldots \left(250\;ns-\Delta t\right)}.$$

Here, *n*_persistent_(*t*, *t* + Δ*t*) is the count of the water molecules within the binding site pocket volume at time *t* that persist in the binding pocket volume at time *t* + Δ*t* based on their having the same unique identifiers. *n*_transient_(*t*, *t* + Δ*t*) is the count of the water molecules within the binding site pocket volume at time *t* that are not in the binding site pocket volume at time *t* + Δ*t* summed with the count of the water molecules that are in the binding site pocket volume at time *t* + Δ*t* but not in the binding site pocket volume at time *t*. The angle brackets indicate an average taken over all *t* in the interval 0 ns to (250 ns—Δ*t*). Identical to the binding site water analysis in the previous subsection, a water molecule was taken to be within the binding site pocket volume if the oxygen atom of that water molecule was located within the sphere radius of any of the pocket alpha spheres computed with fpocket for that snapshot. Based on this definition for the correlation, *C*(Δ*t* = 0 ns) = 1 because *n*_transient_ = 0 and *C*(Δ*t*) = 0 when there is a complete exchange of water molecules in the binding pocket between time *t* and *t* + Δ*t*, that is, when *n*_persistent_ = 0.

**Figure 5:**
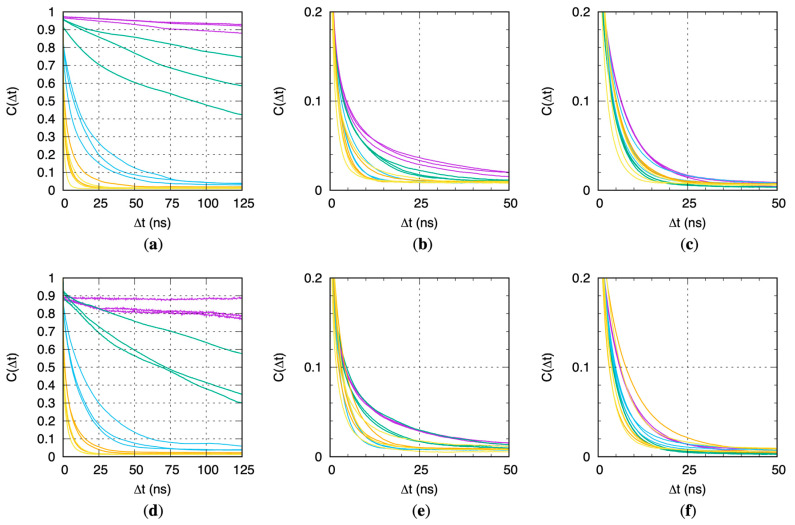
CYP binding site water molecule residence correlations *C*(Δ*t*). Data are for 1A2, 2D6, and 3A4 using (**a**–**c**) the “2 Å” and (**d**–**f**) the “3 Å” solvation protocols. Purple = Stage 1, green = Stage 2, blue = Stage 3, orange = Stage 4.1, yellow = Stage 4.2.

Results of this kinetic analysis are similar to the above equilibrium analysis. In the case of CYP 1A2, there is a dramatic dependence of the kinetics of water exchange on protein flexibility, with greater flexibility promoting more rapid exchange (Figure 5a,d), parallel to greater flexibility promoting equilibrium hydrogen-bond networks between the binding site water and bulk solvent (Figure 2a,d). While similar trends exist for the relationship between increased protein flexibility and increased water exchange kinetics for CYP 2D6 (Figure 5b,e) and CYP 3A4 (Figure 5c,f), they are less dramatic, and there are instances of replicate simulations wherein faster exchange occurs in the restrained (i.e., less flexible) simulation stages (Stages 1–3; purple, green, and blue traces in Figure 5b,c,e,f) compared with the non-restrained simulation stages (Stages 4.1–4.2; orange and yellow traces in the same Figure 5 panels). In strong contrast, for CYP 1A2, the kinetics of water exchange are always in the same order: fully restrained protein with strong positional restraints (Stage 1) < fully restrained protein with weak positional restraints (Stage 2) < C*^α^* atom-only weak positional restraints (Stage 3) < non-restrained protein (Stages 4.1 and 4.2). Taken together, this suggests that the capacity to form a contiguous network of hydrogen-bonded water molecules connecting the binding site and the bulk solvent is a necessary condition for efficient water exchange.

The jitter in the CYP 1A2 Stage 1 traces in Figure 5d arises from the combination of the lack of water exchange with the bulk solvent and the “3 Å” protocol, which results in *n*_persistent_ = 1 and *n*_transient_ = 0 for over 80% of the snapshots across the three replicates. This lone water molecule is the one that is initially ligated to the heme iron and that retains this ligation status for the duration. Per the force field model, this ligation is a non-covalent interaction. That is, there is no bonded term between the water molecule and the iron atom; rather, the association is maintained solely through electrostatic and Lennard-Jones interaction terms. Specifically, the strong electrostatic interaction between the large positive charge of +1.24 at the iron[III] site (see Supplementary Materials) and the large negative charge of −0.834 at the TIP3P water oxygen site [23] ensures persistence of this water molecule as the sixth ligand to the heme iron.

Potentially relevant to the differences observed between 1A2 versus 2D6 and 3A4 may be the crystal structures used as starting conformations for the simulations. As all simulations in this study were of these proteins in their substrate-/inhibitor-free forms, corresponding crystal structures were used if available. While this was possible for 2D6 and 3A4, it was not for 1A2 (see Section 4). Thus, there exists the possibility that the 1A2 results reflect the 1A2 crystal conformation being optimal for binding of the alpha-naphthoflavone ligand present in the 2HI4 co-crystal. As such, removing alpha-naphthoflavone and using the remaining 1A2 protein coordinates to start the simulations may have yielded different results than using 1A2 coordinates from a substrate-/inhibitor-free crystal structure, were such a structure available. In the context of the bound substrate and oxygen, residual water in the active site may lead to uncoupling of the chemistry, which in turn would produce water rather than the desired oxidized product [24,25]. Therefore, the lack of water connection and exchange between the 1A2 binding site and bulk solvent for the conformationally restrained portions (Stages 1–3) of the 1A2 MD trajectories may be a reflection of a protein conformation optimized to take this factor into account. A definitive answer waits on the availability of a substrate-/inhibitor-free crystal structure for human 1A2. We refer interested readers to the discussions of the “solvent channel” and the “water channel” across various CYPs in [18]; access/egress channels in CYPs and controlled exposure of water to the binding site in [19]; and evacuation of water molecules from the 2D6 binding site to accommodate ligand binding in [26].

## 3. Conclusions

If structure-based models of CYP activity can be created to accurately predict drug binding, including both the binding pose and the binding strength, then it will be possible to confidently determine not only if a compound will be bound, but also if it will be oxidized based on the location of the reactive functional groups in the bound pose relative to the CYP heme moiety. For compounds that are predicted to bind but whose binding poses are incompatible with oxidation by the CYP heme, the predicted strength of binding may be correlated with these compounds’ potential to act as CYP inhibitors, which is also an important consideration in drug development. Additionally, knowledge of the inhibitor binding poses can help inform synthetic modifications to convert a drug candidate that is a CYP inhibitor to a new compound that is not as part of the computational drug discovery and design process. Critical to the construction of successful structure-based models is an accurate and complete accounting of the thermodynamically important conformations for a given CYP, because the existence of such a conformational ensemble enables a given CYP to bind a variety of structurally different small molecules [27]. 

Non-restrained microsecond-scale non-biased all-atom explicit-solvent MD simulations, like the ones in the present study, have the potential to enable the enumeration of thermodynamically important protein conformations. However, as with any methodology, possible limitations must be kept in mind. Chief among these are the accuracy of the force field and the completeness of the sampling. With respect to the force field, one factor is the variable performance of different protein force fields, for example, as pertains to modeling well-structured versus flexible or disordered proteins [28,29,30,31]. Another is the choice of water force field parameters, which must be in balance with the protein force field parameters to correctly capture protein–water and water–water interactions but which may not accurately capture the structural and transport properties of the water itself [23,32,33]. A third is the parametrization of the iron[III]-containing heme moiety, which in the present work had parameters assigned by analogy; it may be worthwhile to use quantum mechanics calculations to validate or optimize such parameters. A fourth factor is the lack of dynamic polarizability or charge transfer inherent in fixed-charge force fields, though the use of polarizable force fields does not guarantee that the first three factors will not be issues and comes with additional computational expense [34]. With respect to the completeness of sampling, the timescale of the simulations may pose a limitation. Related to this is the choice of starting conformation, including the necessity to computationally construct missing residues, because the conformations sampled during a simulation may correlate more or less strongly with the starting conformation depending on the timescale of the simulation. Despite these possible limitations, simulations like the ones here offer an opportunity to develop atomic-resolution conformational ensembles beyond what is possible using experimental approaches.

Conformational ensembles of CYP 2D6 from MD simulations have recently been used successfully in the context of machine-learning models of CYP inhibition [11]. In that work, conformations were extracted from 3 ns length trajectories that employed an implicit solvent model. It remains to be seen whether model accuracy could be improved beyond the reported accuracy of 75% by incorporating conformations extracted from microsecond-scale explicit solvent simulations like those in the present work, especially as there exist experimental data to support the idea that human CYPs bind drugs and other substrates predominantly through conformational selection [27]. An open question is whether the simulations used to generate such conformational ensembles ought to incorporate lipid bilayers. CYP crystal structures are bilayer-free because they employ truncated constructs that exclude the transmembrane domain and retain only the globular catalytic domain, whereas the membrane bilayer may affect CYP structure, drug binding, and interactions with redox partners [35]. Recent pioneering work on CYP 2D6 has demonstrated stable microsecond-scale MD simulation of its membrane-anchored form, which was created by combining crystallographic coordinates of the catalytic domain with modeled coordinates for the transmembrane domain embedded in a bilayer [36]. Extension of this work led to the observation of spontaneous ligand binding to CYP 2D6, including facilitated ligand update by an allelic variant known to have increased metabolic activity [26]. It is an open question whether conformational fluctuations in the globular catalytic domain of CYP 2D6 are strongly affected by the presence of the transmembrane domain and its embedding within a bilayer; if they are, it will be important to sample conformations from simulations with lipid bilayers to best incorporate the conformational diversity of a particular CYP during development of a structure-based model. Additionally, the findings regarding the influence of the bilayer on the conformational ensemble of the globular catalytic domain for a given CYP will likely not be generalizable, as it appears that even two CYP proteins in the same subfamily—2C9 and 2C19—can have differing bilayer interactions [37].

## 4. Methods

### 4.1. Molecular Dynamics (MD) Simulations

X-ray crystal structures of the human cytochrome P450 enzymes 1A2 [38], 2D6 [39], and 3A4 [16] were used for starting coordinates for the MD simulations. The PDB [40] IDs for these structures are 2HI4, 2F9Q, and 1TQN, respectively. 2HI4 was chosen because it is the only structure of human 1A2 in the PDB. 2F9Q was chosen because it is the only ligand-free structure of human 2D6 in the PDB. 1TQN was chosen because it is a ligand-free structure of human 3A4, was solved at a better resolution and with fewer missing residues than the previously solved ligand-free structure (PDB ID 1W0E [41]), and was solved at a better resolution than a more recently solved ligand-free structure (PDB ID 4I3Q [42]). Chain A coordinates from each crystal structure were processed with the Reduce software [43] v. 3.23.130521 to add explicit hydrogen atoms and to optimize Asn and Gln sidechain amide group orientations; OH, SH, NH_3_^+^, and methionine methyl rotations; and histidine sidechain protonation states. ROSETTA3 [44] v. 2023.26.351 was used to model the missing loop residues, which were 42–51 for 2F9Q and 282–285 for 1TQN, via the RosettaRemodel functionality [45], and residues immediately flanking the missing residues were also included in the loop modeling. Missing N- and C-terminal residues, that is, those not located in the X-ray experiment, were excluded. Using the CHARMM software [46] v. c45b1, N- and C-termini were constructed in their ionized forms appropriate for neutral pH, and the finalized protein and heme starting coordinates were solvated in cubes of explicit water molecules containing 140 mM NaCl and sufficient additional Cl^−^ ions to generate net neutral systems. First, the cube of water molecules was added, followed by deletion of water molecules that overlapped CYP atoms, and finally, Na^+^ and Cl^−^ ions were added through the random replacement of water molecules located at least 6 Å away from the protein. Prior to solvation, CYP atoms were oriented such that the heme iron was at the origin, the plane of the heme molecule was in the xy plane, and the Cys residue ligated to the heme iron below the xy plane. The cubic systems had edge lengths of *l* + 20 Å, where *l* is length of the longest x-, y-, or z-span of the protein after orientation.

Owing to the buried catalytic pockets of the CYP proteins, two separate solvation protocols were applied to each protein structure to test the effect of the number of water molecules initially within the catalytic pockets on the simulation results. In the first protocol, “2 Å,” water molecules whose oxygen atoms were within 2 Å of any CYP atoms were deleted. In the second protocol, “3 Å,” water molecules whose oxygen atoms were within 3 Å of any CYP atoms were deleted.

MD simulations for each protein were performed in triplicate. For 1A2 (2HI4), starting coordinates for all three simulations were identical. For 2D6 (2F9Q) and 3A4 (1TQN), starting coordinates were the three best-scored RosettaRemodel outputs each. The triplicate simulations combined with the two different solvation protocols meant six independent MD simulation trajectories per CYP. OpenMM [47] v. 8.0.0 was used for all MD simulations, including initial energy minimization and heating. Periodic boundary conditions with a 9 Å cutoff were applied [48], and particle-mesh Ewald was used to account for electrostatic [49] and Lennard-Jones contributions [50] beyond the cutoff. Heating was performed across 0.1 ns from an initial temperature of 31 K, used for assignment of initial randomized velocities, to the target temperature of 310 K using Langevin integration [51] with a friction coefficient of 1 ps^−1^, constant system volume, and harmonic positional restraints of the form *k ×* (Δ*r*)^2^ on all CYP non-hydrogen atoms, where Δ*r* is the displacement from the starting coordinates for a given atom and *k* = 1 kcal·mol^–1^·Å^–2^. This was followed by five successive stages of production MD simulations, all run at 310 K using Langevin integration with a friction coefficient of 0.1 ps^–1^ and at a constant system pressure of 1.01325 bar using a Monte Carlo barostat [52,53]. For Stage 1, the harmonic positional restraints were the same as those used for heating. For Stage 2, *k* was reduced to 0.1 kcal·mol^–1^·Å^–2^. For Stage 3, *k* was maintained at 0.1 kcal·mol^–1^·Å^–2^, but positional restraints were removed from all atoms except for CYP protein C*^α^* atoms. For Stages 4.1 and 4.2, all atomic positional restraints were removed, and a single positional restraint of the same form was applied to the center of mass of the CYP atoms, with *k* = 0.1 kcal·mol^–1^·Å^–2^. The purpose of the center-of-mass restraint was simply to facilitate post-run analysis of the MD snapshots by preventing diffusion of the protein away from the center of the periodic system; without the restraint, each snapshot would need to be re-imaged in order to analyze the CYP–water interactions. Bonds involving hydrogen atoms and water geometries were constrained to their equilibrium values [54,55,56], allowing for the use of a 2 fs integration timestep. Each stage was 250 ns (125 × 10^6^ timesteps) in length, and each successive stage was a continuation of the prior stage, accomplished by using the final atomic positions and velocities from the prior stage, thereby producing a continuous 1250 ns MD trajectory with gradually decreasing atomic positional restraints on the CYP atoms.

Snapshots were taken every 250 ps, resulting in 1000 snapshots per stage. Post-run analysis of snapshots was performed using the CHARMM program, the VMD [57] v. 1.9.4a57-arm64-Rev12 program, the fpocket [15] v. 4.1 program, as well as custom Bash and Python scripts. Crystallographic and MD snapshot conformations used as inputs for fpocket included hydrogen atoms and heme moieties, and fpocket was run with default parameter settings.

### 4.2. Force Field

The CHARMM 36m additive force field for proteins [28,29,30,31] v. jul20 (available at https://mackerell.umaryland.edu/charmm_ff.shtml, accessed on 15 January 2024) was used for all simulations. Water molecules were represented using the TIP3P model [23] modified for the CHARMM force field [58], and Na^+^ and Cl^−^ ion parameters were as described in [59,60]. Force field parameters for the heme with Cys sidechain liganded in the thiolate form were directly transferred from existing parameters, with the following exceptions: the heme iron oxidation state was modeled as iron[III] for consistency with experimental data on CYPs having water as the sixth heme ligand [41], and this required changes to the heme partial charges as well as to the Lennard-Jones parameters for the heme iron atom [61]. Please refer to the Supplementary Materials for the complete set of parameters used in addition to v. jul20.

### 4.3. Root-Mean-Squared Fluctuation (RMSF) Analysis

Atomic RMSF values were first computed on a per-stage basis for each Stage 4.1 and Stage 4.2 portion of each trajectory. All 1000 snapshots from a stage were root-mean-square (RMS) aligned to the starting (crystallographic) conformation using C*^α^* atom positions, and RMSF values for all C*^α^* atoms were computed for this 1000-conformation ensemble. The same procedure was performed a second time, but using for the RMS alignment only those C*^α^* atom positions with a previously computed RMSF value less than 2 Å, so that structural alignment to the reference conformation was not skewed by highly mobile atoms. RMSF values for all C*^α^* atoms and for all sidechain atoms were computed based on this second alignment. A single RMSF value per sidechain was computed by summing the squares of the RMSF values for all atoms in the sidechain, dividing this sum by the number of atoms in the sidechain, and then taking the square root of this sum.

RMSF values were reported in aggregate as residue-wise averages and standard deviations for each of the three CYP proteins. Data aggregation entailed averaging across twelve sets of RMSF data, each from a 1000 snapshot ensemble: (“2 Å” + “3 Å” solvation protocols) × (Stage 4.1 + Stage 4.2) × (triplicate MD simulations).

### 4.4. Binding Site Water Cluster Size

The water cluster size was defined as the number of water molecules within a hydrogen-bond network that included the water molecule ligated to the heme iron atom in the CYP binding site. A “hydrogen bond” was defined as two water molecules having their oxygen atoms within 3.5 Å of each other; that is, each water molecule in the cluster was hydrogen bonded to at least one other water molecule in the cluster. The iron-ligated water molecule was defined as the one having its oxygen atom within 3.5 Å of the heme iron. This water molecule was found to exist in all 30,000 MD snapshots across the six CYP/protocol combinations, with the exception of 4 snapshots: the Stage 1 first snapshot in two runs and the Stage 1 first two snapshots in the third run of the 2D6 simulations using the “3 Å” protocol. Cluster sizes as large as 79 were observed across the 18 MD trajectories, and these corresponded to binding site conformations not connected to the bulk solvent, while the next-smallest observed water cluster size beyond 79 water molecules contained more than 15,000 water molecules (water cluster size = “bulk”), indicative of bulk solvent-connected binding site conformations.

## Figures and Tables

**Table 1:** Crystallographic binding site volumes and residues as determined with fpocket.

CYP	PDB ID	Binding Site Pocket Volume (Å^3^)	Binding Site Residue Numbers ^1^
1A2	2HI4	512	117 118 122 124 125 223 226 227 256 260 312 313 316 317 320 321 382 386 497 498 900
2D6	2F9Q	1019	106 110 112 120 121 175 179 209 210 213 214 216 217 220 244 248 297 300 301 304 305 307 308 309 311 312 370 373 374 482 483 484 486 487 600
3A4	1TQN	2206	50 53 57 76 78 79 105 106 107 108 109 111 115 119 120 121 122 125 212 213 215 216 220 221 223 224 227 230 234 241 301 304 305 308 309 312 369 370 371 372 373 374 481 482 483 484 508

^1^ Last residue number in each list is the heme moiety.

## Data Availability

Data are contained within the article and Supplementary Materials.

## Supplementary Materials

The following supporting information can be downloaded at: https://www.mdpi.com/article/10.3390/molecules29020494/s1, patch file “toppar_all36_prot_heme.str.patch” containing CYP heme-specific topology and parameter information. Apply “toppar_all36_prot_heme.str.patch” to the default “toppar_c36_jul20/stream/prot/toppar_all36_prot_heme.str” file from the CHARMM 36 additive force field v. jul20 available at https://mackerell.umaryland.edu/charmm_ff.shtml, accessed on 15 January, 2024, e.g.: “patch toppar_c36_jul20/stream/prot/toppar_all36_prot_heme.str < toppar_all36_prot_heme.str.patch”.
